# NT-proBNP and Circulating Inflammation Markers in Prediction of a Normal Myocardial Scintigraphy in Patients with Symptoms of Coronary Artery Disease

**DOI:** 10.1371/journal.pone.0014196

**Published:** 2010-12-01

**Authors:** Camilla Noelle Rathcke, Erik Kjøller, Niels Fogh-Andersen, Bo Zerahn, Henrik Vestergaard

**Affiliations:** 1 Department of Internal Medicine, Center of Endocrinology and Metabolism, Copenhagen University Hospital Herlev, Herlev, Denmark; 2 Department of Cardiology, Copenhagen University Hospital Herlev, Herlev, Denmark; 3 Department of Clinical Biochemistry, Copenhagen University Hospital Herlev, Herlev, Denmark; 4 Department of Clinical Physiology and Nuclear Medicine, Copenhagen University Hospital Herlev, Herlev, Denmark; 5 Faculty of Health Sciences, University of Copenhagen, Copenhagen, Denmark; Cuban Neuroscience Center, Cuba

## Abstract

**Background:**

Myocardial perfusion imaging (MPI) can detect myocardial perfusion abnormalities but many examinations are without pathological findings. This study examines whether circulating biomarkers can be used as screening modality prior to MPI.

**Methodology/Principal Findings:**

243 patients with an intermediate risk of CAD or with known CAD with renewed suspicion of ischemia were referred to MPI. Blood samples were analyzed for N-terminal fragment of the prohormone brain natriuretic peptide (NT-proBNP), YKL-40, IL-6, matrix metalloproteinase 9 (MMP-9) and high sensitive C-reactive protein (hsCRP). Patients with myocardial perfusion defects had elevated levels of NT-proBNP (p<0.0001), YKL-40 (p = 0.03) and IL-6 (p = 0.03) but not of hsCRP (p = 0.58) nor of MMP-9 (p = 0.14). The NT-proBNP increase was observed in both genders (p<0.0001), whereas YKL-40 (p = 0.005) and IL-6 (p = 0.02) were elevated only in men. A NT-proBNP cut off-concentration at 25 ng/l predicted a normal MPI with a negative predictive value >95% regardless of existing CAD.

**Conclusions:**

20-25% of patients suspected of CAD could have been spared a MPI by using a NT-proBNP cut-off concentration at 25 ng/l with a negative predictive value >95%. NT-proBNP has the potential use of being a screening marker of CAD before referral of the patient to MPI.

## Introduction

In patients with possible symptoms of coronary artery disease (CAD) it is important not only to detect patients with the disease but at the same time to identify patients with no CAD. Myocardial perfusion imaging (MPI) can be used to demonstrate myocardial perfusion abnormalities in patients with and without known CAD [1]–[3] and to evaluate the risk of new cardiac events in patients with known or intermediate risk of CAD [4], [5]. In daily clinical practice 35–65% of all MPIs are without perfusion defects despite symptoms of myocardial ischemia [6]–[8]. Considering the radiation dose and the significant costs of a MPI, it could be of considerable importance if biomarkers can be used as a screening modality before referral to MPI.

Recently, high sensitivity C-reactive protein (hsCRP) levels have been found in patients with myocardial perfusion abnormalities [9]. CRP is the most examined inflammation marker in relation to cardiovascular disease (CVD) and substantial evidence indicates that baseline hsCRP level is an independent predictor of cardiovascular events both in patients with non-fatal myocardial infarction (MI) and in apparently healthy individuals [10], [11]. Similarly, two recent prospective studies and a meta-analysis of previous studies have shown, that interleukin 6 (IL-6), a proximal mediator of CRP, are associated with risk of CAD about as strongly and in addition to established risk factors [12]. Moreover, the heart failure biomarker N-terminal of the pro-hormone brain natriuretic peptide (NT-proBNP) also has diagnostic and prognostic importance in terms of cardiovascular events and mortality in patients with stable angina pectoris and in patients with acute coronary syndrome [13]–[15]. However, the clinical consequences of elevated NT-proBNP levels are not fully elucidated and concomitantly new markers with different pathophysiological approaches emerge. YKL-40 is a marker of inflammation and endothelial dysfunction, and matrix metalloproteinase 9 (MMP-9) belongs to an enzyme family specialized in breaking down constituents of the extracellular matrix. YKL-40 protein expression is found *in vivo* in both macrophages and vascular smooth muscle cells in the atherosclerotic plaque where it seems to participate in processes during early stages of atherosclerosis by promoting the process of the atherosclerotic plaque formation [16]. The major source for MMPs is also immigrated monocytes/macrophages and vascular smooth muscle cells [17], and MMP-9 seems to be one of the predominant MMPs within the vulnerable plaque, where it promotes plaque progression and destabilization [18], [19]. Both YKL-40 and MMP-9 therefore appear to be associated with the early pathophysiology of atherosclerosis. Furthermore, YKL-40 is associated with the presence and extent of coronary artery disease (CAD) [20]–[22] and elevated YKL-40 levels are seen in patients with myocardial infarction (MI) [22], [23]. Serum MMP-9 levels are gradually increasing with progressing coronary ischemic symptoms [24] and might be useful as an index marker of plaque activity in patients with known CAD [25].

The objective of the present study was to examine whether these markers alone or in combination could be used as a screening modality in patients suspected of CAD prior to referring to MPI.

## Results

Baseline demographic, medical history and paraclinical variables in relation to gender are presented in Table 1. There was an equal distribution of genders and no significant difference in age between genders.

**Table 1:** Clinical characteristics of the study population.

	Total^1^	Male^2^	Female^2^	P value^3^
N	243	118	125	NS
Age*	61.0±11.5	60.9±11.2	61.2±11.9	NS
Smoking	68 (28.0)	39 (33.1)	29 (23.2)	NS
Diabetes	30 (12.3)	14 (11.9)	16 (12.8)	NS
Hypertension	96 (39.5)	43 (36.4)	53 (42.4)	NS
Prior myocardial infarction (MI)	49 (20.2)	31 (26.3)	18 (14.4)	0.002
Prior revascularisation (revasc.)	19 (12.8)	14 (9.7)	5 (2.9)	<0.001
Known CAD (MI or revasc.)	68 (28.0)	45 (38.1)	23 (18.4)	<0.001
**Medication:**				
Beta-blockers	121 (49.8)	66 (55.9)	55 (44.0)	NS
Calcium antagonists	49 (20.2)	24 (20.3)	25 (20.0)	NS
ACE-inhibitors	80 (32.9)	43 (36.4)	37 (29.6)	NS
Diuretics	79 (32.5)	35 (29.7)	44 (35.2)	NS
Statins	111 (45.7)	67 (56.8)	44 (35.2)	<0.001
**Paraclinic:**				
Body Mass Index*	27.7±4.9	28.3±4.3	27.2±5.4	NS
Resting heart rate, bpm*	74.0±12.9	72.1±12.5	75.8±13.1	0.02
Systolic blood pressure, mmHg*	146±23	146±22	147±25	NS
Diastolic blood pressure, mmHg*	87±13	88±13	86±13	NS
Creatinine, µmol/l*	83±21	93±18	75±19	<0.001
Total cholesterol, mmol/l*	5.3±1.3	5.2±1.3	5.4±1.2	NS
HDL, mmol/l*	1.5±0.5	1.3±±0.4	1.7±0.6	<0.001
LDL, mmol/l*	3.0±1.1	3.0±1.2	3.1±1.0	NS
**Stress type at MPI:**				
Ergometer exercise	132 (54.3)	72 (61.0)	60 (48.0)	NS
Dypyridamole	105 (43.2)	43 (36.4)	63 (50.4)	<0.001
Dobutamine stress	6 (2.5)	3 (2.5)	3 (2.4)	NS
**Heart dimensions, post stress:**				
EF, % **	64 (57–70)	59 (51–64)	68 (64–74)	<0.001
EF < lower normal limit***	19 (7.8)	12 (10.2)	7 (5.6)	<0.001
ESV index < lower normal limit, ml/m^2^	0 (0.0)	0 (0.0)	0 (0.0)	NS
**Results of MPI:**				
Normal MPI	199 (81.9)	84 (71.2)	115 (92.0)	<0.001
Abnormal MPI	44 (18.1)	34 (28.8)	10 (8.0)	<0.001

* Mean (SD),

** median (IQR), otherwise presented as N (%).

1 Percentage within the total population,

2 percentage within gender,

3 p value for comparison between genders.

Abbreviations: bpm, beats pr. minute; CAD, coronary artery disease; EF, ejection fraction; ESV index, end systolic volume index; MPI, myocardial perfusion imaging.

There were no differences in systolic and diastolic blood pressure, prevalence of hypertension or diabetes or otherwise use of medication between genders. Although women had higher HDL levels than men, there was no difference in total cholesterol levels. A higher prevalence of CAD was seen in the male part of the population where a higher proportion of treatment with statins also were seen.

An equal number of men and women were capable of performing the bicycle ergometer test, but significantly more women than men were stressed with dypyridamole. Only 19 (7.8%) patients, more men than women, had a low post stress EF but none of these had ESV index below lower normal limit. Myocardial perfusion defects were found in 44 (18.1%) patients, more often in men than in women (p<0.001).

Biomarker levels according to outcome of MPI are shown in Table 2. NT-proBNP levels were elevated in patients with myocardial perfusion defects (p<0.001). This was found for both men and women. YKL-40 and IL-6 levels were significantly elevated in patients with myocardial perfusion defects but only in men (p = 0.005 and p = 0.02, respectively). MMP-9 and hsCRP levels were not elevated in patients with myocardial perfusion defects neither when looking at the genders separately.

**Table 2:** Biomarker levels according to outcome of MPI.

	Normal MPI	Abnormal MPI	p value
**Total study population, N**	**199**	**44**	
NT-proBNP, ng/l	59 (28–143)	264 (109–929)	<0.001
YKL-40, ng/ml	51 (34–80)	67 (40–97)	0.03
IL-6, pg/ml	1.9 (1.2–2.8)	2.2 (1.6–4.0)	0.03
MMP-9, ng/ml	124 (81–183)	146 (82–220)	0.14
hsCRP, mg/l	2.0 (1.4–4.0)	2.2 (1.4–3.8)	0.58
**Male population, N**	**84**	**34**	
NT-proBNP, ng/l	44 (13–82)	279 (102–948)	<0.001
YKL-40, ng/ml	48 (33–71)	67 (51–110)	0.005
IL-6, pg/ml	1.8 (1.3–2.7)	2.3 (1.5–5.1)	0.02
MMP-9, ng/ml	125 (91–174)	147 (84–218)	0.21
hsCRP, mg/l	1.8 (1.4–3.7)	2.2 (1.4–3.7)	0.43
**Female population, N**	**115**	**10**	
NT-proBNP, ng/l	83 (41–167)	226 (124–1330)	<0.001
YKL-40, ng/ml	55 (35–88)	39 (33–66)	0.35
IL-6, pg/ml	1.9 (1.1–2.9)	2.1 (1.6–2.6)	0.86
MMP-9, ng/ml	121 (75–197)	130 (76–268)	0.47
hsCRP, mg/l	2.1 (1.4–4.1)	2.4 (1.4–5.2)	0.97

Presented as median (interquartile range). N, number; MPI, myocardial perfusion imaging; NT-proBNP, N-terminal fragment of the prohormone brain natriuretic peptide; IL-6, interleukine 6; MMP-9, matrix metalloproteinase 9; hsCRP, high sensitive C-reactive protein.

Biomarker levels according to history of CAD are shown in Table 3. NT-proBNP levels were elevated in patients with known CAD (p<0.0001) both in men (p<0.0001) and women (p = 0.003). However, significantly higher NT-proBNP levels were observed in patients with an abnormal MPI when compared to patients with known CAD (264 (109–929) ng/l vs. 111 (54–378) ng/l, p<0.0001). There were not significantly higher biomarker levels of any of the other biomarkers in patients with known CAD when compared to patients without (all p values >0.09).

**Table 3:** Biomarker levels according to known coronary artery disease (CAD; prior myocardial infarction or revascularisation).

	No CAD	CAD	p value
**Total study population, N**	**175**	**68**	
NT-proBNP, ng/l	60 (25–153)	111 (54–378)	<0.001
YKL-40, ng/ml	51 (33–80)	59 (43–97)	0.02
IL-6, pg/ml	1.9 (1.2–2.9)	2.2 (1.3–3.2)	0.27
MMP-9, ng/ml	132 (77–186)	127 (91–189)	0.13
hsCRP, mg/l	2.1 (1.4–4.1)	2.2 (1.4–3.8)	0.43
**Male population, N**	**73**	**45**	
NT-proBNP, ng/l	42 (10–94)	104 (49–488)	<0.001
YKL-40, ng/ml	50 (32–80)	56 (44–86)	0.16
IL-6, pg/ml	1.8 (1.3–3.3)	2.1 (1.4–3.2)	0.71
MMP-9, ng/ml	132 (84–188)	133 (105–183)	0.19
hsCRP, mg/l	1.8 (1.4–3.8)	2.1 (1.4–3.7)	0.92
**Female population, N**	**102**	**23**	
NT-proBNP, ng/l	87 (36–168)	139 (71–300)	0.03
YKL-40, ng/ml	54 (34–80)	64 (38–176)	0.09
IL-6, pg/ml	1.9 (1.1–2.7)	2.3 (1.0–3.5)	0.38
MMP-9, ng/ml	128 (75–183)	100 (80–240)	0.47
hsCRP, mg/l	2.3 (1.5–4.3)	1.7 (1.2–3.2)	0.31

Presented as median (interquartile range). N, number; MPI, myocardial perfusion imaging; NT-proBNP, N-terminal fragment of the prohormone brain natriuretic peptide; IL-6, interleukine 6; MMP-9, matrix metalloproteinase 9; hsCRP, high sensitive C-reactive protein.

**Figure 1:**
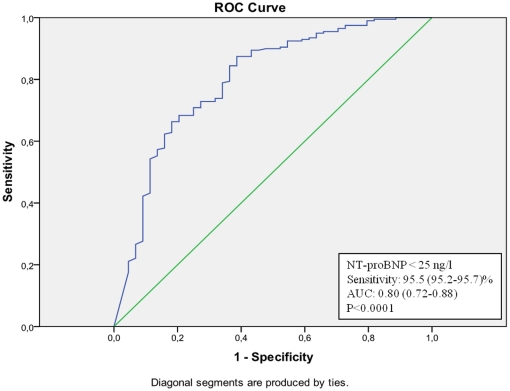
ROC-curve of NT-proBNP values in the prediction of a normal MPI in the total study population.

**Figure 2:**
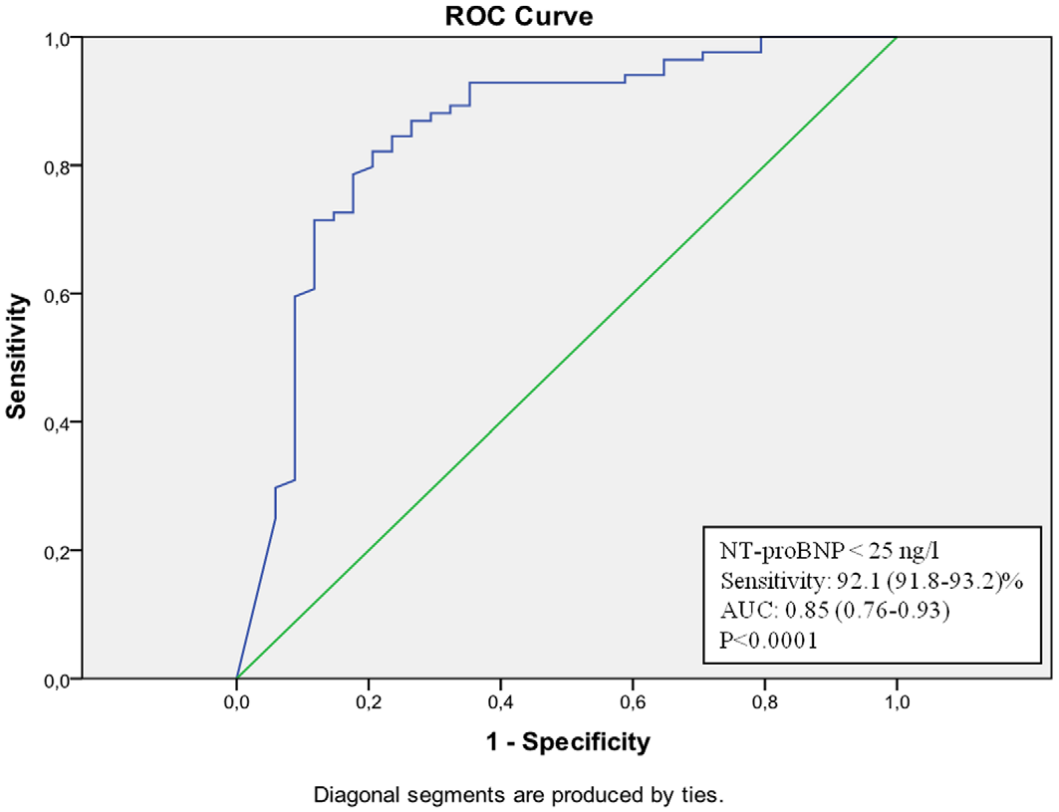
ROC-curve of NT-proBNP values in the prediction of a normal MPI in men (total population).

**Figure 3:**
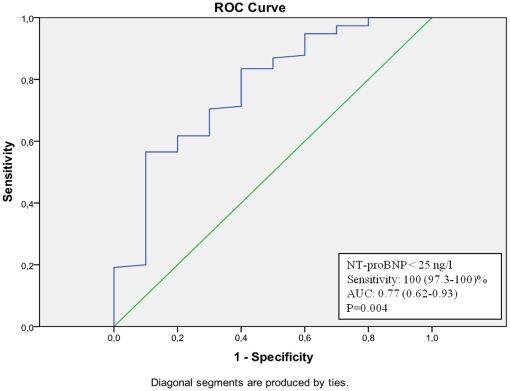
ROC-curve of NT-proBNP values in the prediction of a normal MPI in women (total population).

Using ROC-analyses on NT-proBNP levels for the prediction of a normal MPI, a sensitivity of more than 95.5 (95.2–95.7)% was obtained at a NT-proBNP concentration <25 ng/l in the total population (Figure 1). A total of 42 individuals (21.1%) had a NT-proBNP concentration <25 ng/l but only 2 of these (4.5%) had myocardial perfusion defects. When looking at the genders separately, the sensitivity of a NT-proBNP concentration <25 ng/l as a predictor of a normal MPI was 92.1 (91.8–93.2)% in men (Figure 2) and 100 (97.3–100)% in women (Figure 3). Observed, only 2 of 27 men (7.4%) and 0 of 15 women (0.0%) with a NT-proBNP concentration <25 ng/l had myocardial perfusion defects. Similar ROC curve analyses in subgroups of patients with or without a history of CAD reveal negative predictive values of NT-proBNP 95 ng/ml (sensitivity 96 (95.2–96.9)%) <25 ng/ml (sensitivity 90 (89.3–90.6)%), respectively (curves not shown).

Furthermore, only 5% of the women with a NT-proBNP level <250 ng/l had myocardial perfusion defects equal to a NT-proBNP cut-off concentration of 250 ng/l as a predictor of a normal MPI with a negative predictive value of 95 (84–100)%. Same result is seen in the subgroup of women with known CAD where 4 of 88 women (4.5%) with a NT-proBNP level <250 ng/l had myocardial perfusion defects and that equals a negative predictive value of 95.5 (79.8–100)%.

Univariate regression analyses showed that NT-proBNP levels were correlated with a pathological outcome at MPI (r^2^ = 0.53, p<0.001). This association was not attenuated in multivariate analyses after adjustment for age, gender, smoking, cholesterol level, known CAD, EF, hypertension or diabetes (adjusted r^2^ = 0.57, p<0.001). In univariate analyses, no association was found between myocardial perfusion defects and levels of YKL-40 (p = 0.50), IL-6 (p = 0.19), MMP-9 (p = 0.10) or hsCRP (p = 0.74).

## Discussion

The main results of the present study are that levels of NT-proBNP, YKL-40 and IL-6 are elevated in patients with symptoms of CAD and myocardial perfusion defects. NT-proBNP levels are elevated in both genders whereas levels of YKL-40 and IL-6 are elevated only in men. Using NT-proBNP <25 ng/l as discriminatory value the negative predictive value was >95% with corresponding values for men being 92% and 100% for women. In the subgroup of patients with or without known CAD, the negative predictive value was 96% and 90%. Using a discriminatory value for NT-proBNP <25 ng/l, 21.1% of the MPIs in the total population and 25.3% in patients without known CAD, could have been spared the MPI if relying on NT-proBNP measurement prior to the stress test. It does not seem to be the patients with known CAD who are responsible for this outcome, since patients with myocardial perfusion defects have significant higher NT-proBNP levels. On the basis of these findings, NT-proBNP measurement prior to an MPI could probably be used as a useful gate-keeper test since the finding of few abnormal MPIs suggest a relatively low pre test likelihood for ischemic heart disease in the study population although the population either has CAD or has an intermediate risk of CAD. The difference of the negative predictive value of NT-proBNP between the genders could probably be explained by association between NT-proBNP levels and severity of CAD. It is also well-known, that despite similar risk factors men develop atherosclerosis earlier in life and with a higher incidence than women [26], [27]. However, we have not examined the severity of CAD in the present study, so this remains purely speculative, but we the age between the genders did not differ significantly but we could document 3 times as many men than women with a history of CAD (62% vs. 23%).

Although the predominant pathophysiological process underlying increased circulating levels of NT-proBNP is regional and global impairment of left ventricular systolic or diastolic function leading to increased left ventricular wall stretch, recent studies have suggested that ischemia itself promotes release of BNP [28], [29]. The responsible mechanisms still remain to be fully elucidated, but both experimental and clinical myocardial infarction is associated with gradual and sustained elevation of circulating BNP levels [29] and cardiac BNP expression as verified by cardiac biopsies of hypoxic ventricular areas is of the same magnitude in patients with CAD and with normal left ventricular function as in patients with congestive heart failure but no myocardial ischemia [29]. Furthermore, NT-proBNP has emerged as a potential tool in the diagnosis and therapy of CVD besides heart failure [13], [15], [30]. NT-proBNP concentrations are found to be a prognosticator of long-tem mortality in patients with stable CAD [14], of subsequent MI in patients with unstable CAD [31] and of short term cardiac risk in patients with ACS [32], [33]. Finally, NT-proBNP concentrations below the thresholds used to diagnose heart failure have been found to be associated with an increased mortality risk and risk of cardiovascular events in individuals without heart failure [30].

Our finding of an independent correlation between NT-proBNP and myocardial perfusion defects (reversible and irreversible) supports that high NT-proBNP levels also could be predictive of reversible respectively irreversible myocardial perfusion defects at specific concentration intervals. This is in accordance with a recent epidemiologic study where multiple biomarkers including NT-proBNP substantially improve the risk stratification and prediction of cardiovascular death in individuals with and without CVD [34]. However, this is contradicted by a larger study which investigated the usefulness of NT-proBNP as a predictive marker of angiographic ally significant CAD and CAD severity, where NT-proBNP could not predict significant angiographic lesions following inclusion of traditional risk factors [35]. This objective is investigated further in current studies of our research group.

The overall increased YKL-40 concentration in patients with myocardial perfusion defects was primarily due to elevated YKL-40 levels in men, a difference that cannot be explained by gender differences [36]. Our results are in agreement with previous studies showing that elevated YKL-40 levels are independently associated with the presence [20]–[22] and extent [20] of CAD. Moreover, in patients with MI even higher YKL-levels are documented [22], [23]. YKL-40 has also been found to be associated with all-cause as well as cardiovascular mortality not only in patients with stable CAD [22] but also in the general population above 50 years of age without known diabetes or CAD [37]. In patients with type 1 diabetes, increasing YKL-40 levels are seen with increasing levels of albuminuria as an expression of progressing vascular damages in the kidneys, suggesting that YKL-40 might be used as an early marker of CVD [38]. However, the present findings do not support this hypothesis.

The association between elevated IL-6 levels and myocardial perfusion defects in the present study are in accordance with a meta-analysis where IL-6 levels are associated with risk of CAD [12] but the causality between IL-6 and CAD remains uncertain.

In contrast to the single previous study also designed to examine hsCRP levels in patients referred to a MPI [9], the present study could not document elevated hsCRP levels in patients with myocardial perfusion defects. This divergence could be due to a significantly minor study population (N = 127) with a higher prevalence of men (62%) in the previous study but also due to a study population with less cardiovascular disease. Despite existing knowledge about the role of hsCRP and IL-6 in terms of CAD [11], [12], [18], other studies are not so convincing [10], [39]. Our findings regarding hsCRP and IL-6 are in accordance with a previous study where no association was found between levels of hsCRP or IL-6 and angiographic severity and major cardiac events [39]. In the present study, the explanation for elevated IL-6 levels in men but not in women remains speculative, but might reflect some kind of local production in the heart (whereas CRP is primarily produced by the liver).

We did not find elevated MMP-9 levels in patients with myocardial perfusion defects although MMP-9 is known to destabilize the advanced atherosclerotic plaques [19] and are seen with elevated concentrations in patients with increasing severity of ischemic symptoms [24], [40].

Beside the limitation of being a small-scale study, the foremost limitation is the lack of a pre-test likelihood analysis of the risk of CAD or an abnormal MPI in the study population. One could also dispute the relative high proportion of normal MPIs in this study but in comparison to other studies this is most likely due to the more selected group of participants with less co-morbidity. The advantage of having participants with less co-morbidity is that the NT-proBNP cut-off concentration as a predictor of a normal MPI is strengthened. Furthermore, the small number of participants above 70 years of age (44 individuals of which 13 had an abnormal MPI) make statistical analyses of the influence of age on the predictive value of NT-proBNP obsolete due to lack of statistical power.

We believe the finding of this preliminary study indicates, that NT-proBNP could be used as a screening marker and to be an indicator of a normal MPI when below a certain threshold. This result could be used in the clinical setting in primary care but also at specialized departments of cardiology in the hospital setting before referring a patients to MPI. However, such screening method should never be used alone, but is thought of as a complement in the risk stratification of coronary artery disease and should always be weighed against other risk factors of coronary artery disease. Finally, from a socio-economic perspective the cost of a NT-proBNP analysis is approximately $18, whereas a MPI costs a minimum of approximately $620 but 40% more if pharmacological stress is used (rates at Copenhagen University Hospital Herlev, Denmark). Therefore, beside the patient-orientated clinical aspect, there is also a significant economic aspect which should not be ignored.

This study shows that in patients suspected of CAD, NT-proBNP could possibly demarcate a subgroup of patients without myocardial perfusion defects due to coronary artery stenosis causing reduced blood flow. YKL-40, IL-6, MMP-9 and hsCRP were unable to do so. The prognostic importance of these markers may therefore be their ability to indicate the presence of vulnerable coronary artery plaques, and thereby play an important role in other clinical settings.

The present techniques available to detect the presence/absence of coronary plaques are all either relatively costly, with a not negligible radiation dose or invasive. Although effective, they could appropriately be accompanied by a cheap and easy screening method without major side effects. The clinical implication of the present study is that 20–25% of patients suspected of CAD could have been spared a MPI through the measurement of NT-proBNP in a single blood sample prior to MPI. By using a cut-off concentration of NT-proBNP <25 ng/l and by accepting a negative predictive value of minimum 95% for a normal MPI up to 25% of the normal background population with symptoms of cardiac ischemia referred to a MPI could have been spared this examination.

However, replication of our findings in large-scale studies is needed before using NT-proBNP as a screening marker for normal MPI in the clinical setting. In this context, the importance of inflammation markers like YKL-40, IL-6, MMP-9 and hsCRP should evaluated due to their significant role in the early part of the atherosclerotic process and their ability to detect vulnerable coronary plaques.

## Methods

### Ethics Statement

Informed written consent was obtained from all participants before participation. The study was approved by the local ethics committee of Copenhagen (H-B-2007-058) and investigations conformed to the principles of The Helsinki Declaration.

### Study population

The study population consisted of 243 consecutive referred patients from either a private full time practicing cardiologist or from Dep. of Cardiology, Copenhagen University Hospital Herlev, to an MPI at Dep. of Clinical Physiology, Copenhagen University Hospital Herlev, during the period November 2005 to November 2007.

Patients were referred to MPI either if they were considered to have an intermediate risk of having CAD (symptoms of transient chest pain and/or worsening of chest pain when exercising and/or transient referred pain to the upper limbs or neck) or had a history of CAD with renewed suspicion of ischemia. All participants were clinically examined and included consecutively. Patients with ongoing infectious disease or other concomitant diseases (known chronic obstructive pulmonary disease, cancer, rheumatic or connective tissue disease) as well as patients with reduced renal function (serum creatinine >200 µmol/l) were not included in the study, since inflammation markers, especially YKL-40 levels, are affected in these conditions.

All participants underwent a clinical examination including an ECG and a medical history was obtained and medications were recorded before referral to MPI. The following patient baseline characteristics were registered: History with CAD (previous myocardial infarction (episode with elevated plasma coronary markers and ECG verified myocardial infarction) or coronary revascularization (percutaneous coronary intervention (PCI) and coronary bypass grafting (CABG)), heart failure, hypertension, defined as either systolic blood pressure ≥140 mm Hg, diastolic blood pressure ≥90 mm Hg or use of antihypertensive medicine.

### Measurements

A light caffeine-free meal was allowed prior to MPI. Blood samples were drawn following 15 min of rest in sitting position on the same day as the MPI but prior to this. Routine analyses were performed and the following markers were tested: 1) Plasma NT-proBNP, using a solid double antibody sandwich technique with chemiluminescense as signal (Immulite 2500, Siemens Healthcare diagnostics, Deerfield, IL, USA).Measuring range was <20–35000 ng/l with intra- and interassay coefficients of variation both <5.0% (Low-end reproducibility of NT-proBNP from daily internal quality assessment with low control (nominal concentration 30 ng/L) was 7%, in harmony with the “low level between laboratory precision profile” from UKNEQAS Cardiac Marker External Quality Assessment Service). Siemens Immulite 2500 and Roche Elecsys 2010 use the same assay for NT-proBNP with a mean linear conversion factor between the methods of Y = 0.97 X+26 ng/L, *r* = 0.99; 2) Plasma YKL-40, using an ELISA method (Quidel, USA). Measuring range of the assay was 20–300 ng/ml, with intra- and interassay coefficients of variation of 5.8% and 6.0%, respectively; 3) IL-6, using a high sensitive ELISA (R&D Systems, USA). Lower detection limit was 0.04 pg/ml and intra- and interassay coefficients of variation was 7.4% and 7.8%, respectively; 4) MMP-9, using an ELISA (R&D Systems, USA). Lower detection limit was 0.16 pg/ml and intra- and interassay coefficients of variation was 2.3% and 7.5%, respectively; and 5) CRP, using a highly sensitive, latex-particle-enhanced immunoturbidimetric assay (DAKO, Glostrup, Denmark) with a measuring range of 0.2–80 mg/L and with a lower detection limit of 0.03 mg/L. The results of biomarker analyses were not known when the MPI was done.

### Exercise testing

The stress test was performed as a symptom-limited bicycle exercise test (n = 132). In patients unable to perform physical exercise or did not reach at least 85% of expected heart frequency maximum (220 beats/min – age) a pharmacological stress with dypyridamole/dobutamine was performed according to a standard protocol (n = 111).

The exercise was performed on a bicycle ergometer according to a standard protocol with 25 W increase every second minute. Heart rate, blood pressure and a 12-lead ECG were recorded continuously. A horizontal or down sloping ST-segment depression of at least 1 mm 80 ms after the J-point compared to rest ECG was considered significant for myocardial ischemia. Angina pectoris was defined as chest pain emerging during exercise with relief during recovery or by treatment with nitroglycerin. Injection of 700 MBq^99m^Tc-sestamibi was given at peak exercise level one minute prior to termination of exercise. Criteria for ending the test were fatigue, dyspnoea, angina, ventricular tachycardia, or a decline in systolic blood pressure of more than 20 mmHg during exercise.

### Acquisition and Image Analysis

The myocardial perfusion stress images were obtained one hour after injection. A two-day protocol was applied starting with stress images followed by acquisition of rest images within a week. The images were acquired using a rotating two-headed gamma camera employing a 20% window centred over a photo peak of 140 keV. Patients were scanned in supine position and images were obtained from the right anterior oblique to the left posterior oblique position with 64 steps lasting 20 seconds each and gated acquisition with 12 frames per RR interval. The matrix resolution was 64×64×16 pixels. All images were reviewed by at least two specialists in nuclear medicine with subspecialty in analyses of MPI. No automated-only analyses were used.

Myocardial perfusion was categorized as abnormal if a reversible and/or irreversible perfusion defect was shown involving at least ten percent of the myocardium of the left ventricle and if two or more of 20 segments were hypoperfused more than 2.5 SD below normal. The MPI was also categorized as abnormal when post stress EF and/or end systolic volume (ESV) index were below lower normal limit (EF <51% in men and <43% in women, and ESV index <39 ml/m^2^ in men and <27 ml/m^2^ in women [41]. ESV index was only evaluated in individuals with EF below lower normal limit.

### Statistical analyses

For continuous variables, comparisons between the groups of patients with normal or abnormal MPI were performed by independent samples t-test (Student's t-test), including Levene's test for equality of variance. Mann-Whitney test was used if Levene's test for equality of variance was significant, or if a variable exhibited a clear non-Gaussian distribution. For categorical variables the χ^2^-test was used. Patterns of distribution were examined by histograms and P-Plot analyses. If the distribution is the non-Gaussian a test of statistical (log-) normality was performed.

Concentrations of NT-proBNP, YKL-40, IL-6, MMP-9 and hsCRP were skewed and were logarithmically transformed before further statistical analyses. Subgroup analyses were made for each gender. Since the objectives of the study was to discriminate individuals with a normal MPI and due to an expected low frequency of perfusion defects in this small sample size, analyses of the different types of perfusion defects were mot performed. Data are presented as mean ± SD or as median and interquartile range (IQR).

Analyses of associations were performed using linear regression models with abnormal MPI as dependent variable. Univariate analyses of correlations of either one of the biomarkers NT-proBNP, YKL-40, IL-6, MMP-9 and hsCRP with an abnormal MPI were performed prior to multivariate analyses. Multivariate analyses including age, gender, smoking, cholesterol level, known CAD, EF, hypertension or diabetes were performed for biomarkers with significant outcome in the univariate analyses. ROC-curve analyses of NT-proBNP values for the prediction of a normal MPI was made to assess the NT-proBNP screening cut off-concentration at which a sensitivity of 95% for a normal MPI was achieved. Confidence intervals for sensitivity, specificity and negative predictive values were calculated according to Mercaldo et al [42]. All p-values were calculated as two-sided, and a p-value <0.05 was considered significant. Analyses were made with the statistical software package SPSS® 15.0 (SPSS inc., Chicago, Il, USA) and SAS® 9.0 (SAS, Cary, NC, USA).
